# UPLC-MS/MS profiling, antioxidant and anti-inflammatory activities, and potential health benefits prediction of phenolic compounds in hazel leaf

**DOI:** 10.3389/fnut.2023.1092071

**Published:** 2023-02-01

**Authors:** Jiarui Zhao, Xinhe Wang, Yuchen Wang, Guangfu Lv, He Lin, Zhe Lin

**Affiliations:** College of Pharmacy, Changchun University of Chinese Medicine, Changchun, China

**Keywords:** hazel leaf, phenolics, antioxidant, anti-inflammatory, health benefits

## Abstract

Hazel leaf, one of the by-products of hazelnut, which is widely used in traditional folk medicine around the world. In the present study, the profile of free, conjugated, and bound phenolic compounds from hazel leaf was detected and their antioxidant and anti-inflammatory activities were investigated. The potential health benefits of different phenolic compounds were also predicted. The results showed that the 35 phenolic substances of free, conjugated and bound forms were identified including phenolic acids, flavonoids and catechins. Most of the hazel leaf phenolics were presented in free form, followed by conjugated and bound form. All the fractions effectively inhibited the production of reactive oxygen species and malondialdehyde in TBHP-stimulated human umbilical vein endothelial cells by enhancing endogenous superoxide dismutase, and accordingly alleviated inflammatory cytokines (NO, IL-1β, TNF-α, and IL-6) in LPS-stimulated RAW264.7 cells, showing obvious antioxidant and anti-inflammatory capacity. Moreover, combined with network pharmacology, the potential therapeutic effects and functional pathways of hazel leaf phenolics were predicted, which provided value basis for exploring their treatment on diseases and developing health products in the future.

## Introduction

Hazel belongs to the birch family, which is native to southern Europe, Asia and the American continent and has become an important cash crop in many countries (1). As one of the most popular nuts in the world, hazelnuts are rich in fat, protein, fiber and vitamins, and are consumed in a variety of forms and presentation due to special flavor and sensory properties. Besides, hazelnut skins, hazelnut shells, hazelnut green mulch and hazel leaves are often used in traditional medicine around the world as a by-product from baking, cracking, husking, and harvesting separately (2). In traditional Iranian medicine, hazel leaves are mainly used in the form of infusions as a medicine for liver tonic. Now, they are also used in folk medicine to treat hemorrhoids, varicose veins, phlebitis, and mild edema due to their vascular protection and anti-edema properties (3). Galenic preparations of hazel leaves are also used to relieve ulcers and oropharyngeal infections, and include mild anti-dysentery, anti-fungal, and scarring properties (4). In traditional Swedish medicine, hazelnut leaf and bark are used to treat pain (5). The present studies show that these by-products contain a large amount of phytochemicals, mainly phenolic compounds, which have biological activities such as antioxidant, anti-inflammatory and antibacterial (6). LC-MS method is usually used to analyze the peptides, diarylheptanoids and flavonoids of Corylus species, such as hazelnut, bark, and involucres (7–9). The MS techniques also provide more possibilities for the identification of their constituents and metabolites (10, 11). Among them, the research on hazelnut shells is highly concerned, which contains a large amount of polyphenols, has strong antioxidant and anti-free radical activities, and has inhibitory effects on some tumor cells (12). However, there are few reports on other by-products such as hazel leaves.

Polyphenols are compounds found in plant foods that have potential health-promoting effects. It is found in some common plant foods such as beans, nuts, tea, soya, red wine, vegetables, and fruits (13). As a class of metabolites of natural origin and the largest natural antioxidants in the human diet, polyphenols have direct and indirect antioxidant and anti-inflammatory activities that help to reduce oxidative stress at the cellular level. It has been shown to have anti-bacterial, anti-cancer and other functions that can prevent a range of chronic diseases (14). The polyphenols of perilla leaves demonstrate the antioxidant, anticancer and antidiabetic activities (15). The polyphenolic components in bergamot fruits and leaves have been reported to have good antioxidant and anti-inflammatory properties (16). Oxidative damage and inflammatory are common features of all diseases, and the antioxidant effect of polyphenols extends their application.

Network pharmacology is a new discipline based on the theory of systems biology, which analyzes the network of biological system and selects specific signal nodes to design multi-target drug molecules. It mainly uses various omics, high-throughput screening, network visualization, network analysis and other technologies to analyze and construct drug-target-disease networks by bioinformatics methods, establish prediction models, and analyze the pharmacological mechanism (17). Nowadays, it has been rapidly used in the prediction of constituent targets, the search for active natural products and the discovery of new drugs (18). In many cases, individual food-sourced active compounds are present at very low levels and poor bioavailability, and their therapeutic effects may be limited to food. However, when compounds with similar or complementary effects are combined in the diet, a cumulative effect may occur, which may result in therapeutically effective doses that play an important role in disease prevention (19). Therefore, network pharmacology can also be used to discover which dietary compounds, alone or in combination, have preventive/therapeutic mechanisms for which diseases (20).

The flat-European hybrid hazelnut is a new variety of hazelnut with *Corylus heterophylla* Fisch. as female hazel, *Corylus avellana* L. as male parent since the 1980's (21). So far, the total planting area of flat-European hybrid hazelnut in China is about 50,000 hectares, accounting for more than half of the current planting area (22). This study focuses on flat-European hybrid hazel leaf polyphenols for the first time, investigating its antioxidant and anti-inflammatory effects and potential health benefits prediction. This may provide further insights into hazel leaf as functional food or pharmaceutical additives.

## Materials and methods

### Materials and reagents

The flat-European hybrid hazel leaves were harvested from 7-year-old hybrid hazel trees of local orchard in Jilin Province, China. HPLC-grade acetonitrile and formic acid were purchased from Fisher Scientific (Loughborough, UK). Leucine enkephalin (LE) was supplied by Waters (Milford, USA). All the ultrapure water was prepared with a MilliQ plus water system (Milford, USA).

Human umbilical vein endothelial cells (HUVEC) were purchased from Shanghai Cell Bank, Chinese Academy of Sciences (Shanghai, China). Mouse mononuclear macrophages RAW264.7 were purchased from Guangzhou Saiku Biological (Guangzhou, China). DMEM high sugar medium, Lipopolysaccharide (LPS), 2, 2-diphenyl-1-picrylhydrazyl (DPPH), 2,4,6-Tripyridyltriazine (TPTZ), (+)-Catechin, Gallic acid, Vitamin C, and 6-Hydroxy-2,5,7,8-tetramethylchroman-2-carboxylic acid (Trolox) were purchased from Shanghai Yuanye Biological (Shanghai, China). Mouse TNF-α ELISA Kit, Mouse IL-6 ELISA Kit, and Mouse IL-1β ELISA Kit were obtained from Boster Biological Technology (Wuhai, China). Nitrogen monoxide (NO), Malondialdehyde (MDA), and Total Superoxide Dismutase (SOD) were obtained from Nanjing Jiancheng Institute of Biological Engineering (Nanjing, China).

### Sample preparation

#### Crude extraction of phenolics

Extraction method based on the previous protocol with minor modifications (23). Freeze-dried flat-European hybrid hazel leaves stored in a refrigerator at −80°C were lyophilized and crushed to obtain hazel leaf powder ready for further extraction operations. 1 g of powder was dissolved in 15 mL of 70% methanol solution containing 1% HCl (1–1 by volume), vortexed well and mixed well, and sonicated for 30 min. The mixture was then centrifuged at 1,800 × *g* for 10 min and the extraction was repeated twice. The supernatant was combined to obtain the crude extract. The bottom residue precipitate was stored in a refrigerator at −80°C. The crude extract was removed from the methanol using a vacuum rotary evaporator and the solution was freeze-dried to obtain the crude extract powder.

#### Extraction of free and conjugated phenolics

1 g of crude extract powder was weighed precisely, dissolved in 15 mL of 10 mmol/L hydrochloric acid, extracted with 5 mL of ether-ethyl acetate (1–1 by volume), extracted three times and the organic layers were combined to give free phenol (FP). The combined aqueous layers were subjected to conjugated phenolics extraction by adding an appropriate volume of 6 mol/L sodium hydroxide to a final concentration of 2 mol/L and stirring with a magnetic stirrer for 16 h at room temperature for alkaline hydrolysis. The hydrolysate was acidified to pH 2 with 12 mol/L hydrochloric acid, extracted with ether-ethyl acetate and the organic layers were combined three times to give base-hydrolyzed conjugated phenolics (BCP). The combined aqueous layers were adjusted to a final concentration of 2 mol/L with concentrated hydrochloric acid, hydrolyzed in a water bath at 85°C for 1 h to room temperature, extracted with ether-ethyl acetate and extracted three times to give the combined organic layers as acid hydrolyzed conjugated phenolics (ACP) (24).

#### Extraction of bound phenolics

The bottom residue obtained after centrifugation was freeze-dried, weighed exactly 1 g and redissolved in 15 mL of 2 mol/L of sodium hydroxide solution, mixed thoroughly, sonicated for 30 min and hydrolyzed to insoluble phenolic compounds by gyratory shaking at room temperature for 20 h. The hydrolysate was acidified to pH 2 with 12 mol/L hydrochloric acid, extracted with ether-ethyl acetate and the organic layers were combined three times to give base released bound phenolics (BBP). The remaining aqueous layer was added to an appropriate volume of concentrated hydrochloric acid to the final concentration of 2 mol/L, and hydrolyzed in a water bath at 85°C for 1 h. After cooling to room temperature, the diethylether/ethylacetate (1:1, v/v) was extracted three times and the combined organic layers were acid hydrolysis bound phenolics (ABP). All components are evaporated in portions under nitrogen and stored at −80°C.

### Total phenolic and flavonoid contents

The five fractions were redissolved in 70% methanol (1 mL having 5 mg dry extract) and filtered through a 0.22 μm filter membrane. Total phenolic content (TPC) was determined using the forinol reagent with minor modifications (24). In a 96-well plate, 25 μL of sample or standard was mixed with 125 μL of 0.2 mol/L of forinol reagent for 10 min at room temperature and protected from light, respectively. Then 125 μL of 15% sodium carbonate solution was added and the reaction was carried out at 765 nm for 30 min at room temperature and protected from light for absorbance detection. A standard curve was established using gallic acid standards (50–500 μg/mL) and the TPC was mg gallic acid equivalent (GAE)/g dry weight powder (mg GAE/g DW).

In a 96-well plate, 25 μL of sample or standard was mixed with 110 μL of sodium nitrite (0.066 mol/L) for 5 min at room temperature, 15 μL of 0.75 mol/L aluminum chloride solution was added, followed by 6 min at room temperature. Then 100 μL of 0.5 mol/L sodium hydroxide solution was added and the absorbance was read at 510 nm. A standard curve was established using catechin standards (50–500 μg/mL) and the total flavonoid content (TFC) was given as mg catechin equivalent (CAE)/g dry weight powder (mg CAE/g DW) (25).

### UPLC-TOF-MS/MS analysis

All solvents are filtered through a 0.22 μm filter membrane, five fractions (FP, BCP, ACP, BBP, and ABP) were analyzed in chromatographic fingerprint by the UPLC-TOF-MS/MS system (Waters Q-TOF Synapt G2 high resolution mass spectrometer and Waters ACQUITY UPLC system, Water, Milford, USA) with chromCore 120 C18 column (1.8 μm, 2.1 × 100 mm), equipped with electrospray ionization (ESI). The column temperature was maintained at 40°C, the flow-rate was 0.4 mL/min, and the injection volume was 6 μL. The mobile phase consisted of 0.1% aqueous formic acid (v/v) (A) and acetonitrile (B). Elution was performed with the following gradient: 0–1 min: 5–25% B, 1–5 min: 25–30% B, 5–12 min: 30–50% B, 12–15 min: 50–60% B, 15–20 min: 60–100% B, 20–25 min: 100–5% B. The ESI source was set to negative modes. Nitrogen was used as cone and desolvation gas. The mass spectrometry conditions included desolvation temperature of 500°C, source temperature of 100°C, capillary voltages of 3.0 kV, cone voltages of 35 V, extraction cone voltages of 5.0 V, volume flow rate of 900 L/h and cone gas 50 L/h. The MS^E^ method was applied for MS^2^ analysis. Argon gas was used as collision gas with low collision energy of 5 eV and high collision energy of 25–35 eV.

All MS data were analyzed on the UNIFI platform (Waters, Milford, USA). Using the automatic matching function and database of UNIFI software, compounds can be quickly identified. The parameters were set as follows: the analysis time range is 1–25 min, the mass allowable error range is ±10 ppm, the mass detection range is 50–1,500 Da and the negative adducts containing H^−^ and HCOO^−^. The integrated peak area of high resolution mass spectrometry was used for semi-quantitative analysis.

### Antioxidant assays

#### DPPH assay

As slightly modified from previous practice (26), in a 96-well plate, a standard curve was established using Trolox (62.5–1,000 μmol/L) as the standard. 25 μL of standard or sample (FP, ACP, BCP, BBP, ABP, 1 mL having 5 mg dry extract) was mixed with 200 μL of 350 μmol/L DPPH methanol solution and the absorbance was read at 517 nm after 6 h at room temperature. Antioxidant activity was defined as micromolar Trolox equivalent/gram of dry weight powder (μmol TE/g DW).

#### FRAP assay

Combined the previous FRAP (Ferric-reducing antioxidant power) methods (26), in a 96-well plate, a standard curve was established using L-ascorbic acid (62.5–1,000 μmol/L) as the standard and 10 μL of the standard or sample (FP, ACP, BCP, BBP, ABP, 1 mL having 1 mg dry extract) was mixed with 300 μL of Fe-TPTZ reagent (300 mM acetate buffer: 10 mM TPTZ: 20 mM FeCl_3_- 6H_2_O = 10:1:1) and the reaction was carried out at room temperature for 2 h. The absorbance was read at 593 nm. FRAP values were represented by micromolar ascorbic acid equivalents/g dry weight powder (μmol AAE/g DW).

### Cellular antioxidant activities

#### Cell culture and treatment protocols

Human umbilical vein endothelial cells (HUVEC) were cultured in DMEM with high glycemic complete medium containing 100 U/mL penicillin, 100 mg/mL streptomycin and 10% fetal bovine serum in a 37°C, 5% CO_2_ cell incubator. To elucidate the role of FP, ACP, BCP, BBP, ABP in HUVEC survival, cells were exposed to different FP (4, 8, 12, 16, 20, and 24 μg/mL), ACP (2.5, 5, 7.5, 10, 12.5, and 15 μg/mL), BCP (5, 10, 20, 40, and 80 μg/mL), BBP (5, 10, 15, 20, 25, and 30 μg/mL), and ABP (5, 10, 15, 20, 25, and 30 μg/mL) concentrations over for 24 or 48 h (Supplementary Figures 1A–E). To induce oxidative stress *in vitro*, cells were cultured with a range of concentrations of TBHP (0, 100, 200, 300, 400, and 500 μM), and cell toxicity was measured. After 24 h of administration of TBHP, the cell viability decreased, and 55.8 ± 1.38% of the cell viability was observed at 400 μM (Supplementary Figure 1F). HUVECs were exposed to different phenolics (10 μg/mL) and TBHP (400 μM), and worked together for 24 h to detect the antioxidant capacity.

#### Cell viability assay

The HUVECs were spread in 96-well plates at 10,000 per well. The MTT test was conducted after treatment, 20 μL of MTT (5 mg/mL) was added to each well, incubated for 4 h, carefully sucked out the culture medium, and 150 μL of dimethyl sulfoxide solution (DMSO) was added to each well. The absorbance was read at 490 nm after shaking with an enzyme marker for 10 min.

#### Intracellular reactive oxygen species (ROS) determination

The HUVEC cells were plated at 150,000/mL in 6-well plates, 2 mL per well and 24 h of incubation. TBHP (400 μM) was added to each group except the control group, and then added to the samples. After 24 h, the original medium was discarded for ROS assay. 2, 7-Dichlorofluorescein diacetate (DCFH-DA) was diluted 1:1000 with serum-free high-glucose DMEM medium (1 mL/well), incubated in the incubator for 20 min, washed three times with PBS, and detected by fluorescence microscopy. The fluorescence intensity per unit area calculated by the image analysis system represented the relative level of ROS in cells.

#### Intracellular antioxidant enzyme activities

Groups were cultured as described above. The supernatant of the cellular six-well plate was discarded and washed 2–3 times with phosphate buffered solution (PBS). Each well was added 500 μL PBS and removed the cells with a cell spatula for manual homogenization. The protein concentrations were determined with the BCA Protein Assay Kit, then superoxide dismutase (SOD) and malondialdehyde (MDA) assays were performed according to the kit instructions.

### Cellular anti-inflammatory activities

#### Cell culture and treatment protocols

RAW264.7 mouse macrophages preserved in liquid nitrogen tanks were revived and cultured in DMEM with high sugar complete medium containing 10% fetal bovine serum in a 37°C, 5% CO_2_ cell incubator. To elucidate the role of FP, ACP, BCP, BBP, ABP in RAW264.7 survival, cells were exposed to different FP (10, 20, 30 and 40 μg/mL), ACP (5, 10, 20 and 40 μg/mL), BCP (20, 40, 80 and 160 μg/mL), BBP (6.25, 12.5, 25 and 50 μg/mL), and ABP (6.25, 12.5, 25 and 50 μg/mL) concentrations over for 24 or 48 h (Supplementary Figures 2A–E). RAW264.7 cells were exposed to different phenolics (20 μg/mL) and LPS (1 μg/mL) (27), and worked together for 24 h to detect the anti-inflammatory activities.

#### Cell viability assay

The RAW264.7 cells were spread into 96-well plates at 6,000 per well. After treatment, the MTT assay performed as above.

#### Intracellular anti-inflammatory enzyme activities

The RAW264.7 cells were spread in 96-well plates at the above concentrations and incubated for 24 h. LPS (1 μg/mL) was added to the each group except the control group, and then added to the corresponding samples. After 24 h, the supernatant was carefully aspirated and centrifuged at 2,500 r/min for 10 min. The supernatant was assayed for NO content according to the instructions of the kit, and the TNF-α, IL-6, and IL-1β levels in the cell supernatant according to the instructions of the ELISA kit.

### Prediction of the therapeutic potential and functional pathways of phenolics

The hazel leaf phenolics were fed into the Swiss Target Prediction database for targets prediction. “Oxidative damage” and “inflammation” were filtered in the GeneCards database (https://www.genecards.org/) as disease targets (28). The obtained disease targets were plotted against the phenolics targets in Venny 2.1 (https://bioinfogp.cnb.csic.es/tools/venny/), and the intersecting targets were likely to be the key antioxidant and anti-inflammatory targets of hazel leaf phenolics. The key targets were entered into the STRING (http://string-db.org/cgi/input.pl) online website to construct the protein-protein interaction (PPI) network (28). Network was analyzed and visualized using the Cytoscape 3.9.0. To further understand these targets, the Metascape data platform (https://metascape.org/) was used to perform KEGG pathway enrichment analysis. KEGG enrichment bubble plots were then plotted by Microbiology online platform (http://www.bioinformatics.com.cn/) (29).

### Statistical analysis

The results are presented as means ± SD. All data were analyzed using GraphPad Prism 7.0 software. Differences between groups were analyzed using One-way ANOVA. Tukey's multiple comparisons test analyses were used to calculate statistical differences between multiple groups. The *p*-value < 0.05 was considered statistically significant.

## Results and discussion

### Total phenolic and flavonoid contents

**Figure 1:**
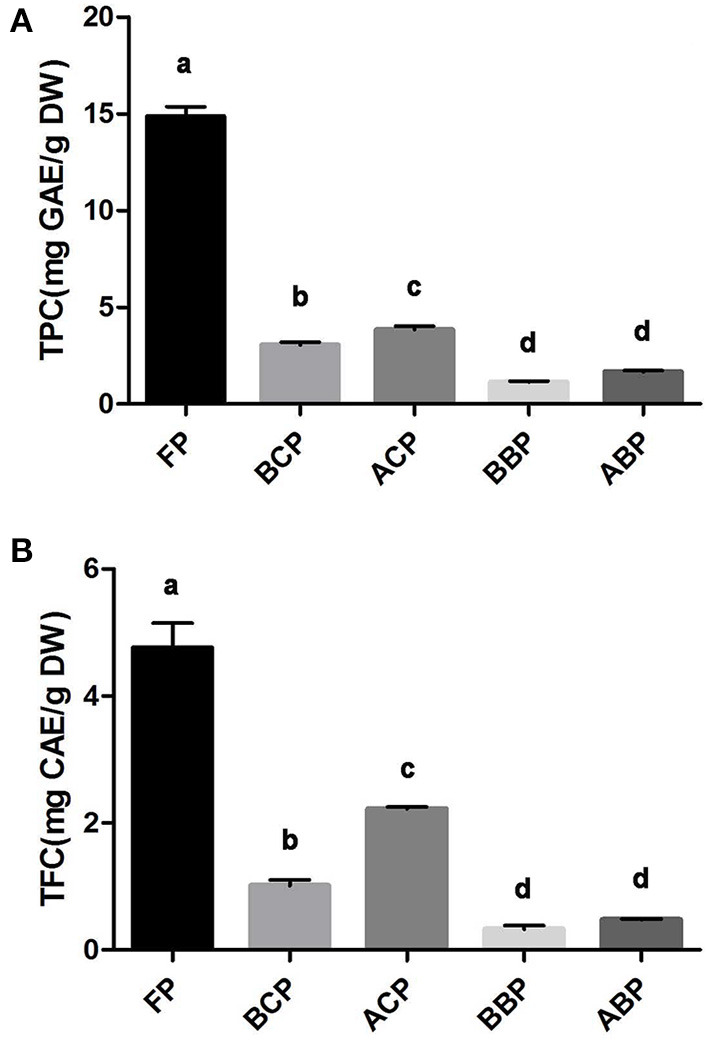
TPC **(A)** and TFC **(B)** in different fractions of hazel leaf, data are presented as means ± SD (*n* = 3), values not sharing a common superscript letter denote significant difference (*p* < 0.05).

The TPC of the different fractions (FP, BCP, ACP, BBP, and ABP) of hazel leaf was shown in Figure 1A, ranging from 1.7 to 14.8 mg GAE/g DW (y = 0.0064x + 0.1517, *R*^2^ = 0.999). The fraction with the highest content was FP, up to 14.8 mg GAE/g DW. The average sum of TPC for all the different phenolic fractions was 24.5 mg GAE/g DW, indicating that hazel leaf are one of the precious options for natural phenolics. And the phenolic content obtained by acid hydrolyses was found to be higher in conjugated and bound phenols than in base hydrolyses extracts. Likewise, the highest TFC was found in FP at 4.76 mg CAE/g DW (Figure 1B). The TFC of five fractions ranged from 0.33 to 4.76 mg CAE/g DW (y = 0.0012x + 0.0403, *R*^2^ = 0.999). The amount of flavonoids released by acid hydrolysis is higher than that of base hydrolyses.

Polyphenols are one of the main classes of plant secondary metabolites. According to their structure, they are divided into phenolic acids, flavonoids, lignans, stilbenes, and curcumins (30). Phenolic acids include benzoic acid derivatives (or hydroxybenzoic acid) and cinnamic acid derivatives (or hydroxycinnamic acid). Benzoic acid derivatives are mainly found in fruits and vegetables in conjugated form (esters or glycosides), but also in free form. Hydroxycinnamic acid rarely exists in free form, but mainly in conjugated form. The main dietary flavonoids are flavanols and proanthocyanidins, which are mostly in the form of monomers and polymers (31). Since they exist in different forms, either free or bound, this feature needs to be fully considered in the extraction process in order to improve the yield. The literature on TPC in hazelnut kernel and byproducts mainly focuses on hazelnut, hazelnut shell and hazelnut skin, and only a very limited amount has been isolated in hazel leaf (32–34). The hazel leaf used in this study was from hybrid hazelnut, which is the first report of different forms of phenolic compounds in this variety. Furthermore, considering the potential role of conjugated and bound phenolics in body health, especially gut health, it is important to closely examine the composition of different phenolic fractions.

### Characterization of free, conjugated, and bound phenolics

**Figure 2:**
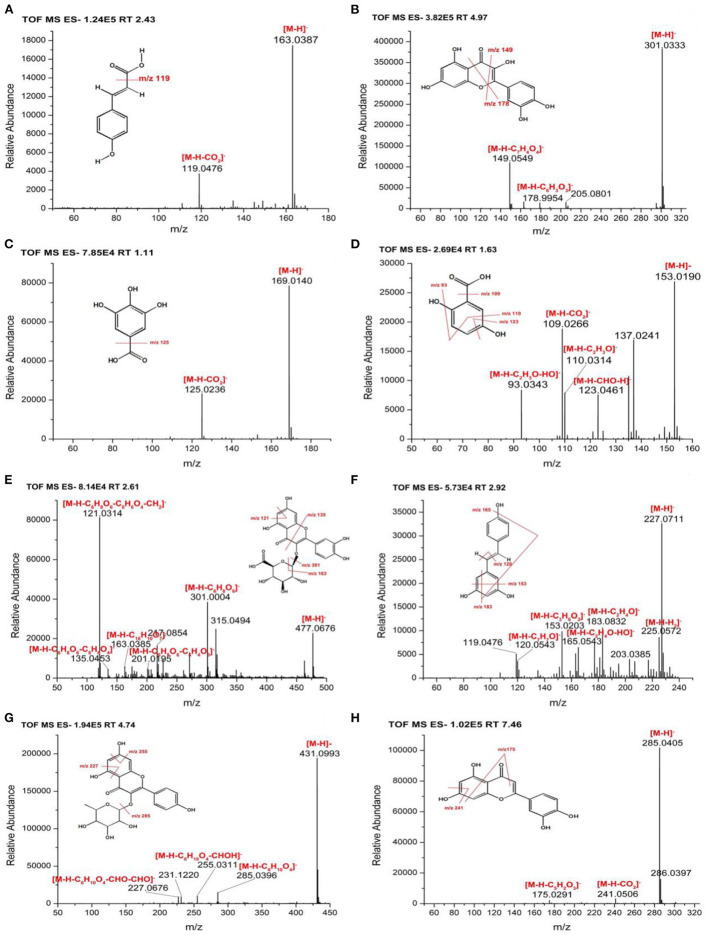
MS/MS spectra and fragmentation patterns of selected phenolic compounds **(A)** p-coumaric acid, **(B)** quercetin, **(C)** gallic acid, **(D)** 2, 5-dihydroxybenzoic acid, **(E)** quercetin-3-*O*-beta-D-glucuronide, **(F)** resveratrol, **(G)** kaempferol-3-*O*-rhamnoside, and **(H)** luteolin.

In this study, the TOF-MS/MS method was used to characterize phenolic compounds in different parts of hazel leaf (FP, BCP, ACP, BBP, and ABP). Based on retention time, MS data, fragment ions, molecular weights, literature and databases, a total of 35 phenolic compounds had been tentatively identified in free, esterified and bound forms as shown in Table 1. In the FP fraction, FP1 was tentatively identified as gallic acid with a precursor ion at *m/z* 169.0128 and fragment ions at *m/z* 125.0236 ([M–H–CO_2_]^−^) (35). FP2 ([M–H]^−^, *m/z* 305.0641) was tentatively identified as gallocatechin based on fragment ions at *m/z* 109.0220 ([M–H–C_9_H_8_O_5_]^−^), 137.0241 ([M–H–C_8_H_8_O_4_]^−^), 179.0306 ([M–H–C_6_H_6_O_3_]^−^), 219.0535 ([M–H–C_4_H_6_O_2_]^−^), and 261.0735 ([M–H–CO_2_]^−^) as the reported MS/MS data (36). FP3 showed a precursor ion at *m/z* 153.0182 and a major fragment ion at *m/z* 109.0266 ([M–H–CO_2_]^−^), and was tentatively identified as 2, 5-dihydroxybenzoic acid (37). FP4 ([M–H]^−^, *m/z* 353.0846) with fragment ions at *m/z* 191.0518 ([M–H–C_5_H_6_O_6_]^−^), 192.0529 ([M–H–C_5_H_5_O_6_]^−^), and 161.0183 ([M–H–C_11_H_12_O_3_]^−^) was tentatively identified as chlorogenic acid (36). FP5 gave a precursor ion at *m/z* 463.0847 and a major fragment ions at *m/z* 300.9928 ([M–H–glycoside]^−^) corresponding to quercetin-3-*O*-beta-D-glucopyranoside (38). FP6 was tentatively identified as p-coumaric acid with a precursor ion at *m/z* 163.0388 and a major fragment ion at *m/z* 119.0476 ([M–H–CO_2_]^−^), and details were shown in Figure 2A (35). FP7 ([M–H]^−^, *m/z* 317.028) was tentatively identified as myricetin based on fragment ions at *m/z* 165.0543 ([M–H–C_8_H_8_O_3_]^−^), 179.0658 ([M–H–C_7_H_6_O_3_]^−^), 288.1270 ([M–H–CHO]^−^), and 315.0105 matching with data in the Pubmed databases. FP8 ([M–H]^−^, *m/z* 315.0492) showed fragment ions at *m/z* 271.0560 ([M–H–CO_2_]^−^), 301.0004 ([M–H–CH_2_]^−^), and 311.0933, and was tentatively characterized as pedalitin matching with data in the Pubmed databases. FP9 was tentatively identified as quercetin with a precursor ion at *m/z* 301.0333 and fragment ions at *m/z* 149.0549 ([M–H–C_8_H_8_O_3_]^−^) and 178.9954 ([M–H–C_7_H_7_O_2_]^−^) as shown in Figure 2B (37). FP10 ([M–H]^−^, *m/z* 447.0902) was tentatively identified as luteolin-7-*O*-glucoside with fragment ions at *m/z* 285.1414 ([M–H–C_9_H_6_O_3_]^−^) and 327.1232 ([M–H– C_7_H_4_O_2_]^−^) (39).

**Table 1:** Identification and relative content of individual phenolics in free, conjugated, and bound forms in hazel leaf.

**Peak**	**RT** **(min)**	**Formula**	**[M–H]^−^** **(*m/z*)**	**Major fragment ions (*m/z*)**	**Error** **(ppm)**	**Tentative identification**	**Relative content** **( × 10^4^)**
**Free phenolics (FP)**							
FP1	1.00	C_7_H_6_O_5_	169.0128	125.0236	−8.3	Gallic acid	2.35 ± 0.28
FP2	1.37	C_15_H_14_O_7_	305.0641	109.0220, 137.0241, 179.0306, 219.0535, 261.0735	−8.6	Gallocatechin	2.79 ± 0.57
FP3	1.50	C_7_H_6_O_4_	153.0182	109.0266	−7.1	2,5-dihydroxybenzoic acid	0.72 ± 0.02
FP4	1.68	C_16_H_18_O_9_	353.0853	191.0518, 192.0529, 161.0183	−7.1	Chlorogenic acid	4.13 ± 0.21
FP5	2.35	C_21_H_20_O_12_	463.0847	300.9928	−7.5	Quercetin-3-*O*-beta-D-glucopyranoside	34.02 ± 6.77
FP6	2.43	C_9_H_8_O_3_	163.0388	119.0476	−8.1	p-Coumaric acid	10.57 ± 2.83
FP7	2.61	C_15_H_10_O_8_	317.0280	165.0543, 179.0658, 288.1270, 315.0105	−7.2	Myricetin	67.37 ± 11.70
FP8	3.11	C_16_H_12_O_7_	315.0492	271.0560, 301.0004, 311.0933	−5.9	Pedalitin	4.23 ± 0.91
FP9	4.97	C_15_H_10_O_7_	301.0333	149.0549, 178.9954	−6.8	Quercetin	86.44 ± 11.06
FP10	9.20	C_21_H_20_O_11_	447.0902	285.1414, 327.1232	−6.8	Luteolin-7-*O*-glucoside	48.19 ± 12.32
**Base-hydrolysable conjugated phenolics (BCP)**							
BCP1	1.11	C_7_H_6_O_5_	169.0140	125.0236	−1.6	Gallic acid	8.42 ± 0.72
BCP2	1.63	C_7_H_6_O_4_	153.0190	93.0343, 109.0266, 110.0314, 123.0461	−2.5	2,5-dihydroxybenzoic acid	3.38 ± 0.66
BCP3	2.30	C_9_H_8_O_4_	179.0351	135.0453	0.5	Caffeic acid	9.94 ± 1.25
BCP4	2.61	C_21_H_18_O_13_	477.0676	121.0314, 135.0453, 163.0385, 201.0195, 301.0004	0.3	Quercetin-3-*O*-beta-D-glucuronide	15.12 ± 2.52
BCP5	2.92	C_14_H_12_O_3_	227.0711	120.0543, 153.0203, 165.0543, 183.0832, 225.0572	−1.4	Resveratrol	4.59 ± 0.52
**Acid-hydrolysable conjugated phenolics (ACP)**							
ACP1	1.06	C_7_H_6_O_5_	169.0130	125.0236	−1.3	Gallic acid	2.97 ± 0.56
ACP2	3.06	C_9_H_8_O_3_	163.0391	119.0476, 120.0543, 121.0266, 135.0453, 145.0306	−5.8	p-Coumaric acid	1.19 ± 0.43
ACP3	4.48	C_15_H_10_O_8_	317.0297	165.0190, 287.0119, 288.9984, 315.0183	−2	Myricetin	2.38 ± 0.54
ACP4	5.86	C_15_H_10_O_6_	285.0401	241.0591	−1.1	Luteolin	1.33 ± 0.45
ACP5	5.91	C_15_H_10_O_7_	301.0344	149.0042, 179.0013	−3.3	Quercetin	28.30 ± 5.33
ACP6	8.72	C_14_H_6_O_8_	300.9978	185.9775, 257.0444, 299.1262	−4	Ellagic acid	15.79 ± 4.37
**Base-released bound phenolics (BBP)**							
BBP1	1.24	C_7_H_6_O_5_	169.0142	125.0236	−0.3	Gallic acid	6.24 ± 0.92
BBP2	2.04	C_7_H_6_O_3_	137.0245	93.0343	0.5	Hydroxybenzoic acid	4.46 ± 0.56
BBP3	2.35	C_9_H_8_O_4_	179.0351	135.0453, 136.0457	0.8	Caffeic acid	4.33 ± 0.82
BBP4	2.56	C_21_H_20_O_12_	463.0891	301.0004	2	Quercetin-3-*O*-beta-D-glucopyranoside	2.73 ± 0.84
BBP5	2.74	C_21_H_18_O_13_	477.0686	121.0314, 201.0195, 299.0185, 301.9977	2.3	Quercetin-3-*O*-beta-D-glucuronide	6.43 ± 0.60
BBP6	2.97	C_9_H_8_O_3_	163.0400	119.0524, 120.0543	−0.6	p-Coumaric acid	4.57 ± 0.63
BBP7	4.12	C_21_H_20_O_11_	447.0939	285.0396, 327.0915	1.4	Luteolin-7-*O*-glucoside	24.88± 5.72
BBP8	4.74	C_21_H_20_O_10_	431.0993	227.0676, 255.0311, 285.0396	2.3	Kaempferol-3-*O*-rhamnoside	27.77± 5.36
**Acid-released bound phenolics (ABP)**							
ABP1	1.00	C_7_H_6_O_5_	169.0140	125.0236	−1.7	Gallic acid	11.32 ± 3.74
ABP2	1.68	C_7_H_6_O_4_	153.0188	95.0452, 108.0172, 109.0266, 123.0072	−3.8	2,5-dihydroxybenzoic acid	1.68 ± 0.58
ABP3	1.94	C_7_H_6_O_3_	137.0242	79.0213, 93.0343, 109.0311	−1.3	Hydroxybenzoic acid	1.79 ± 0.74
ABP4	4.61	C_15_H_10_O_8_	317.0298	165.0148, 288.9526, 315.0183	−1.5	Myricetin	4.36± 0.84
ABP5	6.17	C_15_H_10_O_7_	301.0352	151.0042, 178.9954	−0.6	Quercetin	40.17 ± 7.89
ABP6	7.46	C_15_H_10_O_6_	285.0405	175.0291, 241.0506	0.1	Luteolin	19.96 ± 5.77

In the BCP fraction, BCP1 was already detected and identified in the FP fraction as gallic acid (Table 1, Figure 2C). BCP2 ([M–H]^−^, *m/z* 153.019) was tentatively identified as 2, 5-dihydroxybenzoic acid with fragment ions at *m/z* 93.0343 ([M–H–C_2_H_3_O–HO]^−^), 109.0266 ([M–H–CO_2_]^−^), 110.0314 ([M–H–C_2_H_3_O]^−^), and 123.0461 ([M–H–CH_2_O]^−^) as shown in Figure 2D (37). BCP3 showed a precursor ion at *m/z* 179.0351 and a major fragment ion at *m/z* 135.0453 ([M–H–CO_2_]^−^), and was tentatively identified as caffeic acid (36). BCP4 ([M–H]^−^, *m/z* 477.0676) was tentatively characterized as quercetin-3-*O*-beta-D-glucuronide based on fragment ions at *m/z* 121.0314 ([M–H–C_6_H_8_O_6_-C_8_H_6_O_4_-CH_2_]^−^), 135.0453 ([M–H–C_6_H_8_O_6_-C_8_H_6_O_4_]^−^), 163.0385 ([M–H–C_16_H_10_O_7_]^−^), 201.0195 ([M–H–C_6_H_8_O_6_-C_4_H_4_O_3_]^−^) and 301.0004 ([M–H–C_6_H_8_O_6_]^−^), and the details were shown in Figure 2E (40). BCP5 ([M–H]^−^, *m/z* 227.0711) with fragment ions at *m/z* 120.0543 ([M–H–C_7_H_7_O]^−^), 153.0203 ([M–H–C_3_H_6_O_2_]^−^), 165.0543 ([M–H–C_2_H_4_O–HO]^−^), 183.0832 ([M–H–C_2_H_4_O]^−^), and 225.0572 was tentatively characterized as resveratrol matching with data in the Pubmed databases (Figure 2F).

In the ACP fraction, ACP1 and ACP5 were already detected and identified in the FP fraction as gallic acid and quercetin (Table 1). ACP2 ([M–H]^−^, *m/z* 163.0391) with fragment ions at *m/z* 119.0476 ([M–H–CO_2_]^−^), 120.0543 ([M–H–CH_3_-CO]^−^), 121.0266 ([M–H–CO–CH_2_]^−^), 135.0453 ([M–H–CO]^−^), and 145.0306 ([M–H–H_2_O]^−^)was tentatively characterized as p-coumaric acid (35). ACP3 ([M–H]^−^, *m/z* 317.028) was tentatively identified as myricetin with fragment ions at *m/z* 165. 0190 ([M–H–C_8_H_8_O_3_]^−^), 287.0119 ([M–H–CH_2_O]^−^), 288.9984 ([M–H–CHO]^−^), and 315.0183 matching with data in the Pubmed databases. ACP4 was tentatively identified as luteolin with a precursor ion at *m/z* 285.0401 ([M–H]^−^) and a major fragment ion at *m/z* 241.0591 ([M–H–CO_2_]^−^) (41). ACP6 ([M–H]^−^, *m/z* 300.9978) gave fragment ions at *m/z* 185.9775 ([M–H–C_4_H_3_O_4_]^−^), 257.0444 ([M–H–C_2_H_3_O]^−^), and 299.1262 corresponding to ellagic acid (36, 42).

In the BBP fraction, BBP1, BBP4, BBP6, and BBP7 were already detected and identified in the FP fraction as gallic acid, quercetin-3-*O*-beta-D-glucopyranoside, p-coumaric acid and luteolin-7-*O*-glucoside (Table 1). BBP2 showed a precursor ion at *m/z* 137.0245 and a major fragment ion at *m/z* 93.0343 ([M–H–CO_2_]^−^), and was tentatively characterized as hydroxybenzoic acid (38). BBP3 and BBP5 were already detected and identified in the BCP fraction as caffeic acid and quercetin-3-*O-*beta-D-glucuronide (Table 1). BBP8 with a[M–H]^−^ ion at *m/z* 431.0993 and fragment ions at *m/z* 227.0676 ([M–H–C_6_H_10_O_4_-2CHO]^−^), 255.0311 ([M–H–C_6_H_10_O_4_-CHOH]^−^), and 285.0396 ([M–H–C_6_H_10_O_4_]^−^) was tentatively characterized as kaempferol-3-*O*-rhamnoside, and details were shown in Figure 2G (43).

In the ABP fraction, ABP1, ABP2, ABP3, ABP4, and ABP5 were already detected and identified in the FP, BCP and BBP fraction as gallic acid, 2,5-dihydroxybenzoic acid, hydroxybenzoic acid, myricetin, and quercetin (Table 1). ABP6 ([M–H]–, *m/z* 285.0405) showed fragment ions at *m/z* 175.0291 ([M–H–C_6_H_6_O_2_]^−^) and 241.0506 ([M–H–CO_2_]^−^), and was tentatively identified as luteolin (Figure 2H) (41).

Based on our preliminary identification and relative content analysis of phenolic compounds, FP had the most kinds of compounds, with 10 compounds. The component with the highest mass spectrum peak area was myricetin, followed by quercetin-3-*O*-beta-D-glucopyranoside and p-coumaric acid. The BBP had eight components, of which the highest peak area was luteolin-7-*O*-glucoside and kaempferol-3-*O*-rhamnoside. Both ACP and ABP had six components, among which quercetin had the highest peak area. The BCP had five components, of which the highest peak area was Quercetin-3-*O*-beta-D-glucuronide. The total peak area of FP polyphenols was 6.37 times that of BCP, and 5.02, 3.3, 3.22 times that of ACP, ABP, and BBP respectively. Gallic acid was found in all five fractions, which is consistent with the report that five phenolic acids (gallic acid, caffeic acid, p-coumaric acid, ferulic acid, and erucic acid) were identified and quantified from hazelnut leaves, and gallic acid was the most in free and esterified forms (2). Quercetin and myricetin were found in 3 fractions. Similarly, phenolic compounds including myricetin, quercetin, kaempferol, and related derivatives were also identified in a comparative study of the leaves of 10 different hazelnut varieties from northeastern Portugal (44). Differently, Rosmarinic acid, Hirsutenone 3-caffeoylquinic acid, caffeoyltartaric acid, and oregonin were detected in the leaves of *Corylus avellana* L. and *Corylus maxima* Mill. by LC-MS method (4, 45, 46). In addition, some amino acids including valine, alanine, GABA, aspartic acid and tyrosine were detected in *Corylus avellana* L. leaves by NMR method (47).

### Antioxidant capacity

#### DPPH and FRAP assays

**Figure 3:**
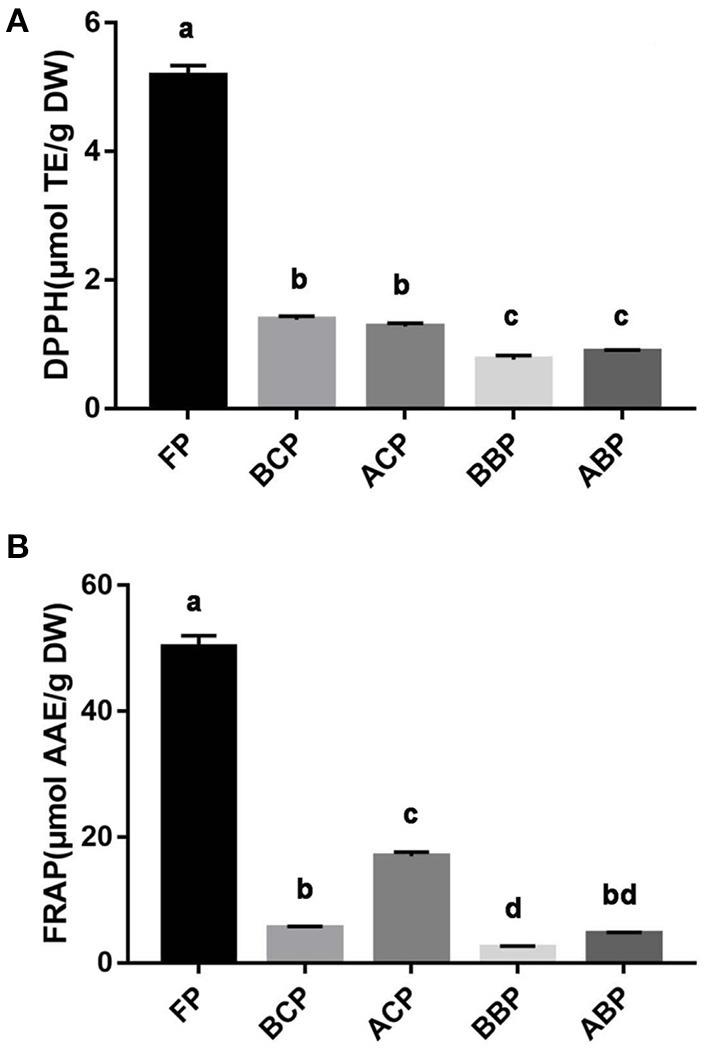
Antioxidant capacity of different fractions of hazel leaf **(A)** DPPH assay, **(B)** FRAP assay, data are presented as means ± SD (*n* = 3), values not sharing a common superscript letter denote significant difference (*p* < 0.05).

The phenolic extracts of hazel leaf were briefly tested for chemical redox capacity by *in vitro* chemical assays. DPPH and FRAP assays are commonly used *in vitro* chemical methods to assess antioxidant capacity. DPPH radical has a strong absorption capacity. When antioxidants are paired with the single electron of DPPH radical, the change of absorbance at 517 nm is often used as an indicator to measure the scavenging effect of DPPH radical (26). Fe-TPTZ is reduced to the ferrous form by reducing species, which has a maximum light absorption at 593 nm. The antioxidant activity intensity of the samples can be calculated based on the absorbance (26). As shown in Figures 3A, B, the DPPH and FRAP method were performed with Trolox and L-ascorbic acid as the standard and calibration curve y = −0.0022x + 1.2136, *R*^2^ = 0.999 and y = 0.002x + 0.0487, *R*^2^ = 0.998, respectively. According to the results, it was found that free phenolics had the highest radical scavenging activity, followed by conjugated phenolics (BCP and ACP) and bound phenolics (BBP and ABP). The antioxidant capacity of different fractions of hazel leaf was basically consistent with the contents of TPC and TFC, which also verified that phenolics were the main substances of antioxidant activity.

### Cellular antioxidant capacity

**Figure 4:**
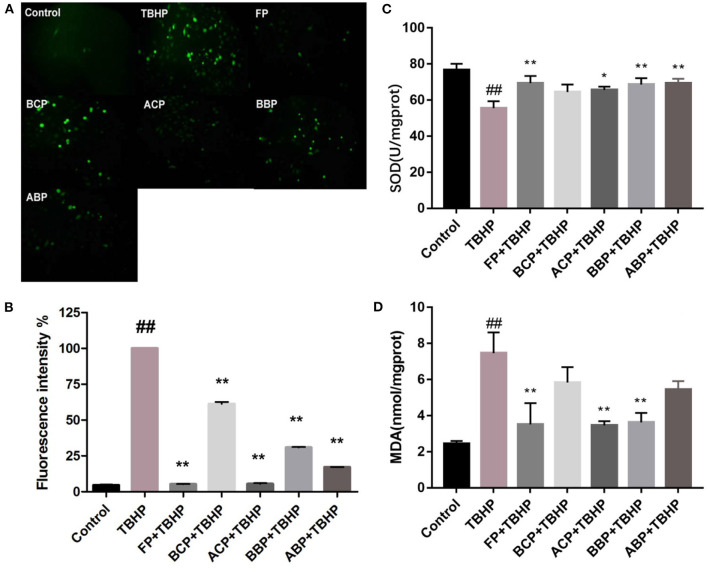
Influence of hazel leaf phenolics on ROS **(A, B)**, SOD **(C)**, and MDA **(D)** in TBHP-stimulated HUVEC cells, values are expressed as mean ± SD, ^##^*p* < 0.01 vs. Control, **p < 0.01 vs. TBHP, *p < 0.05 vs. TBHP.

Reactive oxygen species (ROS) are the main forms of free radicals present and are indicative of the extent of oxidative damage to cells, which include hydroxyl radicals, hydrogen peroxide radicals, and superoxide. Excess ROS can disrupt the balance between oxidative and antioxidant systems in cells, leading to oxidative stress, which further damage the structure and function of cells (48). The results of fluorescent microscope showed that compared with the control group, the green highlights in the TBHP group were significantly increased, indicating that the cell oxidative damage was established successfully. Compared with the TBHP group, the green highlights of phenolic extracts were significantly reduced, indicating that the different fractions of hazel leaf have antioxidant properties, but the BCP group was weaker. The intensity of green fluorescence was quantified based on image analysis system. The results showed that the fluorescence intensity of each fraction group was significantly lower than the TBHP group, and the FP and ACP groups was close to the control group relatively, indicating a strong antioxidant capacity. Moreover, the other three groups also had good antioxidant capacity, which was consistent with the fluorescence microscope observation (Figures 4A, B).

The level of SOD activity indirectly reflects the body's ability to scavenge oxygen free radicals, while the level of MDA reflects the severity of free radical attack on the body's cells (48). As shown in Figures 4C, D, compared with the control group, the SOD level in the TBHP group decreased significantly (*p* < 0.01). Compared with the TBHP group, FP, ACP, BBP, and ABP increased significantly (*p* < 0.05, *p* < 0.01), and BCP was slightly higher but not statistically significant. Meanwhile, the MDA content in the TBHP group was markedly higher than the control group (*p* < 0.01). The content of FP, ACP, and BBP group were markedly lower than the TBHP group (*p* < 0.05, *p* < 0.01), and BCP and ABP were slightly lower but not statistically significant. The results indicated that phenolic extracts could alleviate oxidative damage by improving SOD activity and MDA content in cells, among which FP, ACP, and BBP were quite effective.

### Cellular anti-inflammation capacity

**Figure 5:**
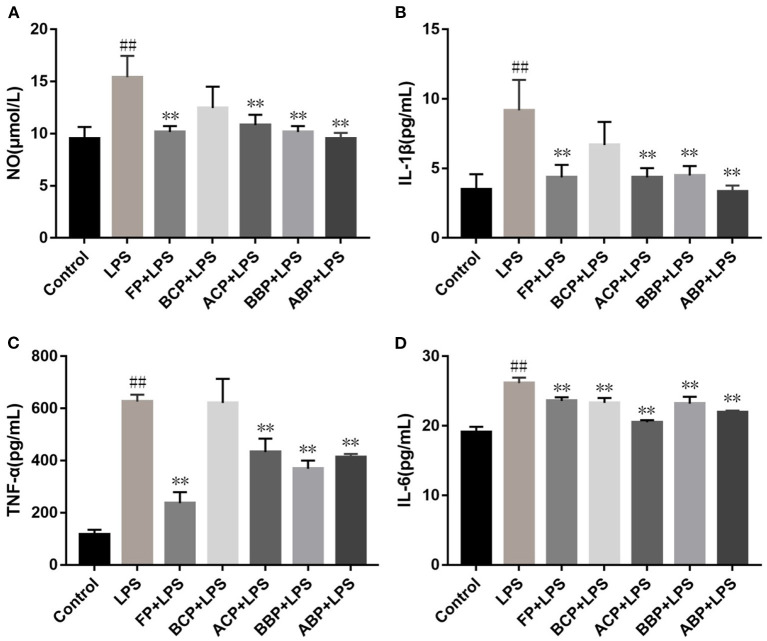
Influence of hazel leaf phenolics on NO **(A)**, IL-1β **(B)**, TNF-α **(C)**, and IL-6 **(D)** in LPS-stimulated cells, values are expressed as mean ± SD, ^##^*p* < 0.01 vs. Control, **p < 0.01 vs. LPS.

Exogenous LPS stimulation makes cells produce NO, and the accumulation of excess NO stimulates neutrophils and macrophages to release pro-inflammatory factors such as TNF-α, IL-1β, and IL-6. These proinflammatory factors have a wide range of biological effects and also induce the expression of other proinflammatory factors, which play an important role in the initiation of inflammation and immune responses (49). As shown in Figures 5A–D, compared with the control group, the levels of NO, IL-1β, TNF-α, and IL-6 in the LPS-stimulated group were significantly increased (*p* < 0.01). Compared with the LPS group, the NO, IL-1β, TNF-α, and IL-6 level of FP, ACP, BBP, and ABP group reduced clearly (*p* < 0.01). For the BCP group, compared with the LPS group, the intracellular IL-6 level was significantly decreased, and the NO, IL-1β, and TNF-α decreased but no significant difference. The results showed that phenolic extracts could alleviate the inflammatory response by reducing the accumulation of NO in LPS-induced cells and inhibiting the release of inflammatory factors TNF-α, IL-1β, and IL-6. Among the different forms of phenolic extracts, FP, ACP, BBP, and ABP were quite effective, which were basically consistent with the antioxidant capacity.

### Prediction of the therapeutic potential of phenolics

The phenolic compounds of hazel leaf obtained by UPLC-TOF/MS were screened by the Swiss ADME platform, and 17 active constituents were retained and imported into the Swiss Target Prediction database to obtain the targets. After screening and deduplication, a total of 591 targets were obtained. The active ingredients and targets data above were imported into Cytoscape 3.9.0 for network construction. Base on the components-targets network analysis, the closer the relationship between active ingredients and targets with higher degrees of freedom, the more likely they have potential therapeutic effects. The top three active ingredients were Quercetin-3-O-beta-D-glucopyranoside, luteolin and Quercetin, with degree values of 191, 164, and 118 respectively, which are presumed to be important components of hazel leaf phenolics (Table 2).

**Table 2:** Active ingredients of hazel leaf phenolics.

**No**.	**Component name**	**Degree**
1	2,5-dihydroxybenzoic acid	8
2	Hydroxybenzoic acid	26
3	Gallic acid	26
4	Gallocatechin	20
5	p-Coumaric acid	33
6	Caffeic acid	27
7	Chlorogenic acid	115
8	Quercetin	118
9	Ellagic acid	64
10	Resveratrol	60
11	Quercetin-3-O-beta-D-glucuronide	105
12	Kaempferol-3-O-rhamnoside	73
13	Luteolin	164
14	Luteolin-7-O-glucoside	84
15	Pedalitin	105
16	Quercetin-3-O-beta-D-glucopyranoside	191
17	Myricetin	104

**Figure 6:**
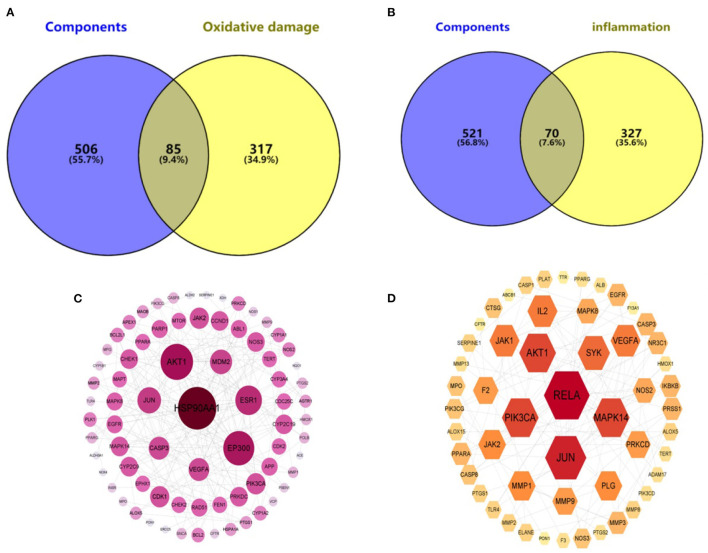
Component—oxidative damage **(A)**/inflammation **(B)** target distribution map and PPI network of oxidative damage **(C)**/inflammation **(D)**.

In the Genecards database, 402 oxidative damage-related targets and 397 inflammation-related targets were obtained. They were intersected with 591 targets of the hazel leaf phenolics, resulting in 70 and 85 intersecting targets, respectively, as shown in Venn diagrams (Figures 6A, B). The common targets of oxidative damage and inflammation were interacted through the PPI network and visualized by Cytoscape 3.9.0, with a total of 77 nodes and 210 edges for oxidative damage, and 53 nodes and 141 edges for inflammation. In the PPI network, the importance of the targets was shown by the size of the degree value normally. The eight targets with degree values ≥10 were HSP90AA1, AKT1, EP300, ESR1, JUN, MDM2, CASP3, and VEGFA in the oxidative damage PPI network (Figure 6C). The six targets were RELA, JUN, AKT1, PIK3CA, MAPK14, and SYK in the inflammation PPI network as well (Figure 6D). It was hypothesized that these targets might play an important role in the antioxidative and anti-inflammation of hazel leaf phenolics.

**Figure 7:**
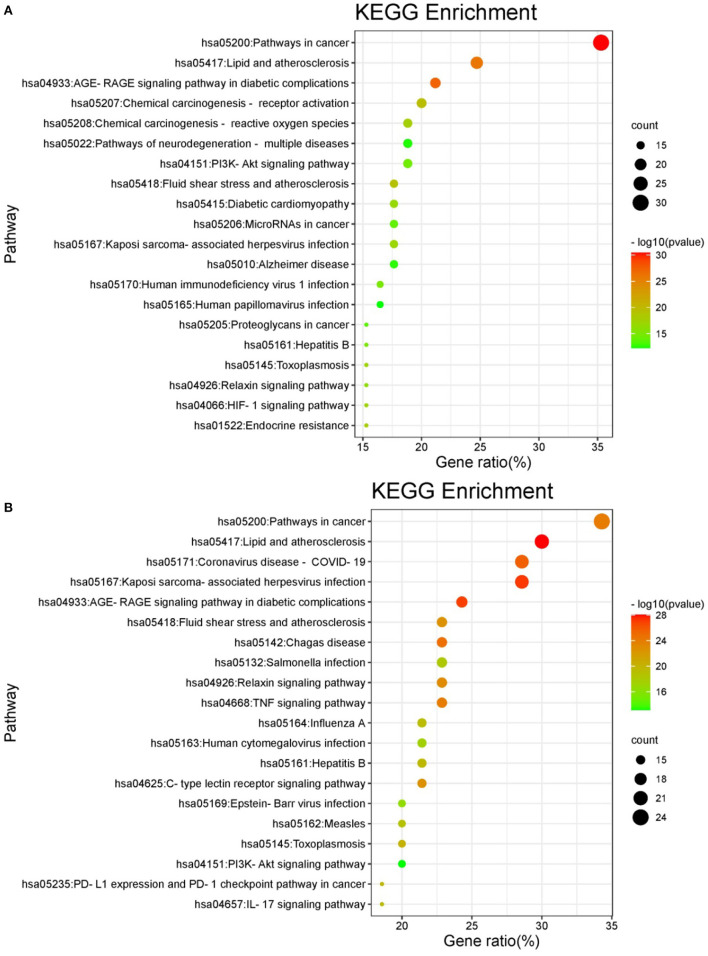
KEGG pathway enrichment of oxidative damage **(A)**/inflammation **(B)**.

KEGG enrichment analysis was performed for these intersecting targets. A total of 162 signaling pathways related to oxidative damage (*P* < 0.01) and 150 signaling pathways related to inflammation (*P* < 0.01) were obtained. Among them, the top 20 pathways with the highest probability were selected for visualization. As shown in the bubble enrichment diagram, the more genes enriched in the pathway, the bigger the bubble, the smaller the *P*-value and the redder the color. The oxidative damage-related targets were mainly involved in cancer, lipid and atherosclerosis, diabetic complications, chemical carcinogenesis, and neurodegenerative disease pathways (Figure 7A). The inflammation-related targets were mainly involved in cancer, lipid and atherosclerosis, coronavirus disease, herpesvirus infection, and diabetes complications pathways (Figure 7B). Based on the network pharmacological prediction of antioxidant and anti-inflammatory effects of hazel leaf phenolics, three important core targets were the same, namely JUN, AKT1, and VEGF. KEGG enrichment analysis also showed that they shared functional pathways targeting cancer, lipids and atherosclerosis, as well as diabetes complications. These results indicated that hazel leaf phenolics may have potential therapeutic effects on cancer, obesity, cardiovascular disease and diabetes through the signaling pathways involved in the above key targets, such as the MAPK pathway (50, 51). It narrowed the potential therapeutic scope of hazel leaf phenolics and the corresponding mechanism, which would be helpful for its in-depth understanding and utilization. However, the definite conclusion still needs further experimental and clinical confirmation.

## Conclusion

This study investigates comprehensive information on phenolics in free, conjugated and bound forms of flat-European hybrid hazel leaf. Our results provide the first detailed analysis of hazel leaf phenolics and confirm that hazel leaf phenolics are mainly present in free form. Gallic acid is the most distributed phenols, which has been identified in all forms of components. Pedalitin, chlorogenic acid, and gallocatechin were only found in free phenols, resveratrol and ellagic acid were only found in conjugated phenols, and kaempferol-3-O-rhamnoside and hydroxybenzoic acid were only found in bound phenols. As determined by chemical and cell viability, the FP fraction had the highest antioxidant and anti-inflammatory activities, followed by ACP, BBP, ABP, while BBP had the weakest activity. Combined with polyphenol content and characterization analysis, this result may be positively correlated with the composition of phenolic components. Furthermore, with network pharmacology, we predicted the potential therapeutic effects of hazel leaf polyphenols on cancer, lipids, atherosclerosis and diabetic complications, and detected the corresponding functional pathways. This work provides an overall picture of phenolic profile in hazel leaf, expand the plant sources of dietary polyphenols and facilitate the further design functional food formulations.

## Data availability statement

The raw data supporting the conclusions of this article will be made available by the authors, without undue reservation.

## Author contributions

HL and ZL: research design. JZ and XW: research implementation. YW and GL: data analysis. HL: writing and editing. All authors read and approved the final manuscript.
