# Supplementary material for: UPLC-MS/MS profiling, antioxidant and anti-inflammatory activities, and potential health benefits prediction of phenolic compounds in hazel leaf
Source: Front Nutr. 2023 Feb 1;10:1092071. doi: 10.3389/fnut.2023.1092071 (PMC9929368; doi:10.3389/fnut.2023.1092071)
Supplement: Supplementary file 1 [file Data_Sheet_1.PDF]

Supplementary Material

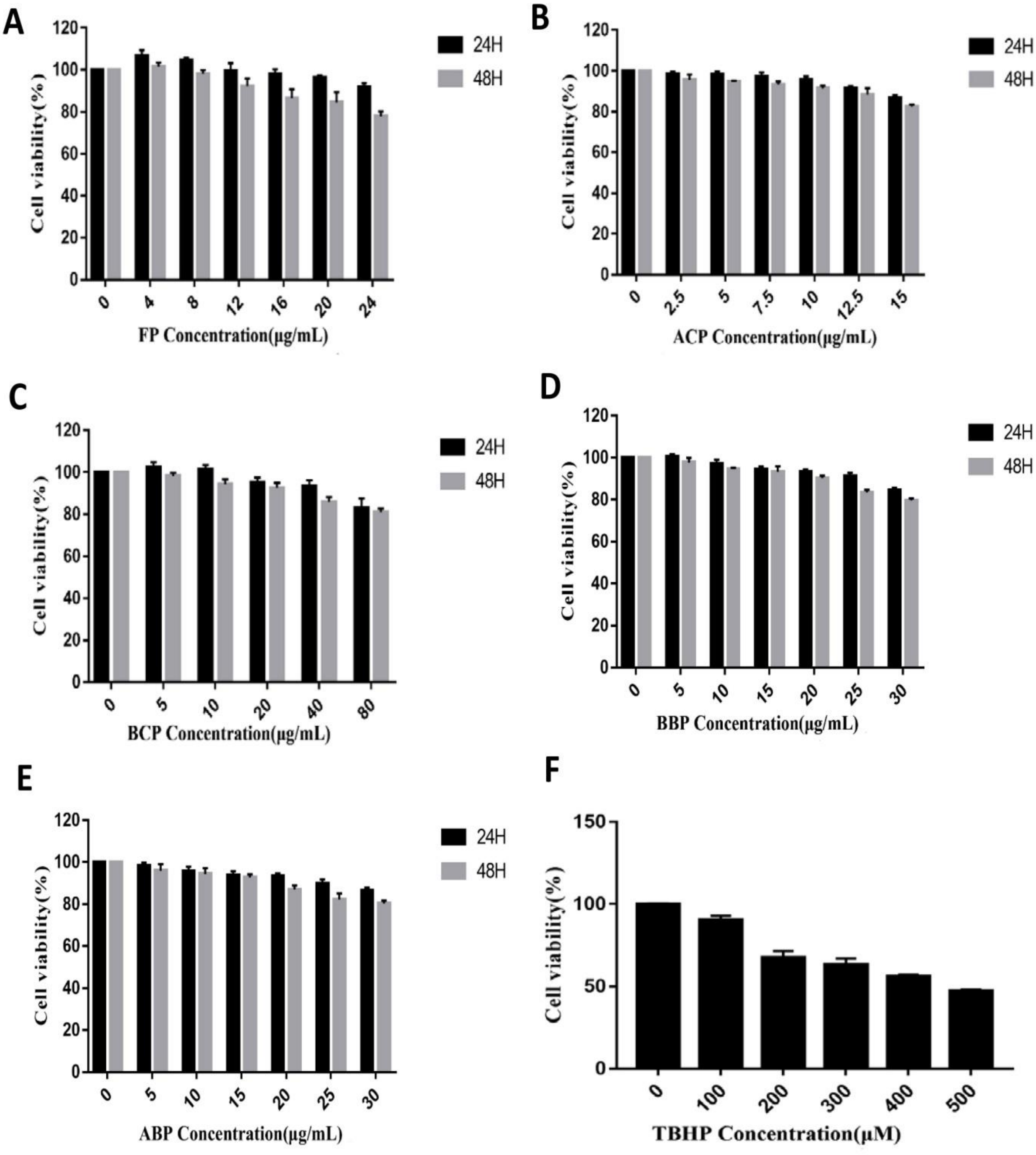

Figure S1 Evaluation of HUVEC viability using MTT assay after exposure to different concentrations of phenolics for 24 h or 48h and different concentrations of TBHP for 24 h, FP (A), ACP (B), BCP (C), BBP (D), ABP (E), TBHP (F).

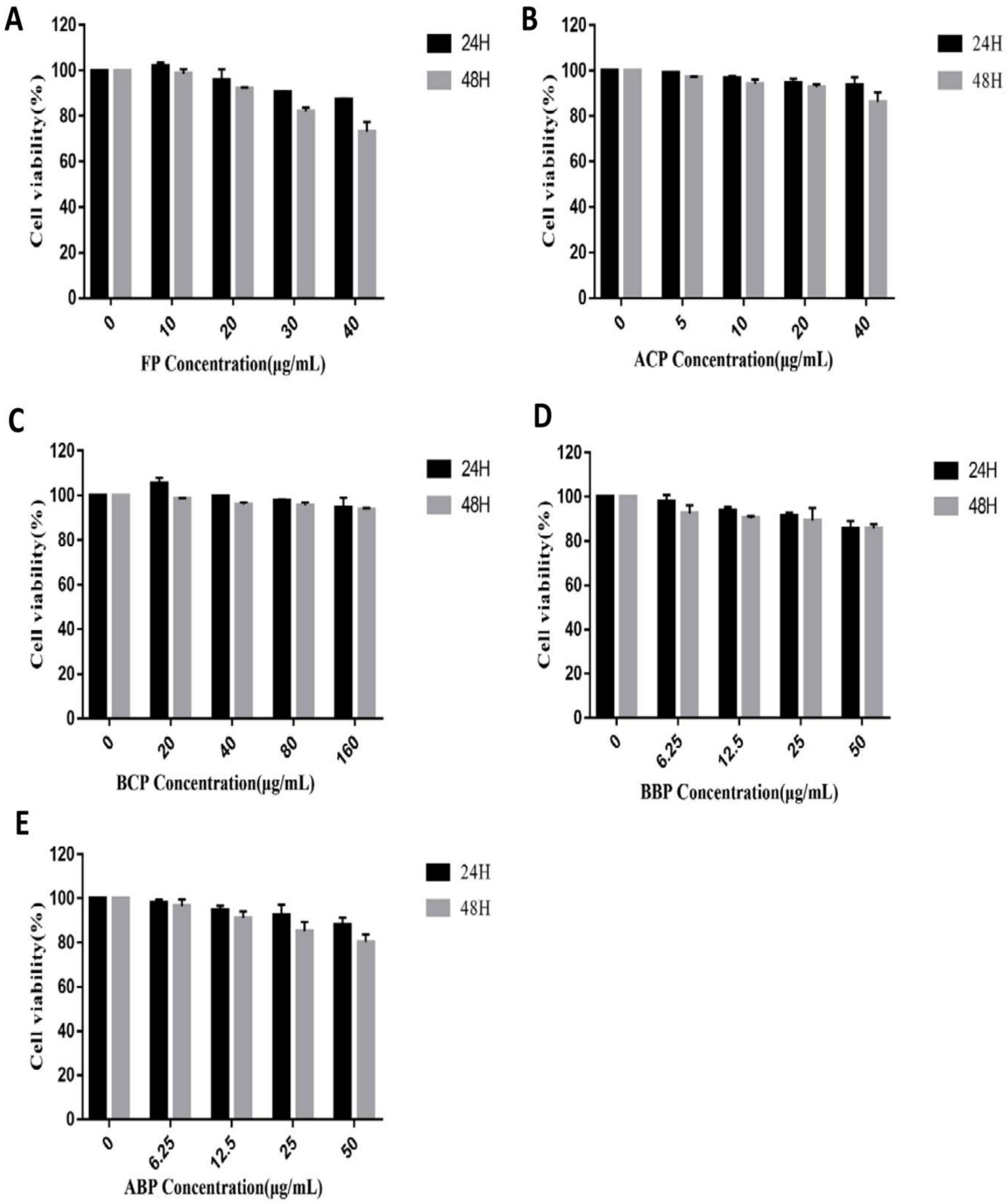

Figure S2 Evaluation of RAW264.7 viability using MTT assay after exposure to different concentrations of phenolics for 24 h or 48h, FP (A), ACP (B), BCP (C), BBP (D), ABP (E).
